# Effects of L1 Transfer Are Profound, Yet Native-Like Processing Strategy Is Attainable: Evidence From Advanced Learners’ Production of Complex L2 Chinese Structures

**DOI:** 10.3389/fpsyg.2021.794500

**Published:** 2021-12-03

**Authors:** Fuyun Wu, Jun Lyu, Yanan Sheng

**Affiliations:** ^1^School of Foreign Languages, Shanghai Jiao Tong University, Shanghai, China; ^2^Department of Linguistics, University of Southern California, Los Angeles, CA, United States; ^3^School of Foreign Studies, Shanghai University of Finance and Economics, Shanghai, China

**Keywords:** L2-Chinese, processing strategy, determiner positioning, relative clause, L1-transfer

## Abstract

English as a verb-medial language has a short-before-long preference, whereas Korean and Japanese as verb-final languages show a long-before-short preference. In second language (L2) research, little is known regarding how L1 processing strategies affect the ultimate attainment of target structures. Existing work has shown that native speakers of Chinese strongly prefer to utter demonstrative-classifier (DCL) phrases first in subject-extracted relatives (DCL-SR-N) and DCLs second in object-extracted relatives (OR-DCL-N). But it remains unknown whether L2 learners with typologically different language backgrounds are able to acquire native-like strategies, and how they deviate from native speakers or even among themselves. Using a phrase-assembly task, we investigated advanced L2-Chinese learners whose L1s were English, Korean, and Japanese, because English lacks individual classifiers and has postnominal relative clause (RC), whereas Korean and Japanese have individual classifiers and prenominal RCs. Results showed that the English and Korean groups deviated from the native controls’ asymmetric pattern, but the Japanese group approximated native-like performance. Furthermore, compared to the English group, the Korean and Japanese groups favored the DCL-second configuration in SRs and ORs. No differences were found between the Korean and Japanese groups. Overall, our findings suggest that L1 processing strategies play an overarching role in L2 acquisition of asymmetric positioning of DCLs in Chinese RCs.

## Introduction

To attain native-like performance, a second language (L2) learner needs to adopt new strategies in a target language while suppressing competition from her first language (L1). This is a challenging task because languages differ in their syntactic and typological properties, and thus their processing strategies also vary. For instance, in determining the subject/agent of a sentence, English as a strict subject-verb-object (SVO) language relies heavily on word order (e.g., MacWhinney et al., 1984; MacDonald, 1987a), French as a language with rich inflections utilizes verbal agreement (Heilenman and McDonald, 1993), and Chinese as an isolating language with little morphology gives priority to animacy over SVO word order (Miao et al., 1986; Su, 2001). When processing strategies of L1 are in conflict with those of a target L2, linguistic transfer often occurs across the board (lexicon: Poulisse, 1999; De Groot and Keijzer, 2000; Jiang, 2002; Jarvis, 2009; (morpho-)syntax: White, 1985; Jin, 1994; Su, 2001; Wakabayashi, 2002; Ionin et al., 2004; Yuan and Zhao, 2005; Tolentino and Tokowicz, 2011; Hopp, 2017; Hopp and Grüter, 2021; discourse/pragmatics: Williams, 1988; Granger and Tyson, 1996; Zufferey et al., 2015), especially during an early stage of acquisition (Bates and MacWhinney, 1981; Kilborn and Ito, 1989; Sasaki, 1991, 1994; Liu et al., 1992; Su, 2001). Sometimes, L1 transfer can also be observed in advanced L2 learners. As reported in Su (2001), even advanced Chinese-speaking L2-English learners relied heavily on animacy, rather than on word order, when determining the grammatical functions of Animate Noun + Inanimate Noun + V (NNV) and V + Animate Noun + Inanimate Noun (VNN).

However, other studies have documented a gradual shift from L1 strategies to L2 strategies (MacDonald, 1987b), with some even showing that L1 processing strategies can be overridden given sufficient L2 proficiency (Dussias, 2003; Dussias and Sagarra, 2007; Tuninetti et al., 2015). For instance, through extensive exposure to English, Dutch-speaking L2-English learners showed evidence of relinquishing case inflection as a cue when identifying the agent in various constructions, including datives, NVNs, and relative clauses (RCs) (MacDonald, 1987b). Results of such kind have led some researchers to claim that target-L2 processing strategies are ultimately attainable (MacWhinney, 2005, p. 77).

It is worth noting that most L2 studies investigating L1 transfer of parsing strategies demonstrated by advanced learners have focused on comprehension, with few probing language production, particularly at the sentence level. To our knowledge, one study that directly addresses the question of whether L2 learners can fully attain native-like production strategies is Dennison (2008), focusing on phrasal ordering preference. In verb-medial languages such as English, short phrases tend to be uttered before long or heavy phrases (Wasow, 1997; Clark and Wasow, 1998; Stallings et al., 1998; Arnold et al., 2000). For instance, compared to a bare noun (“home”), a complex noun phrase (NP) modified by an RC is most likely to be shifted to the end, as in *I invited home a friend that I missed very much*, rather than the other way around (*I invited a friend that I missed very much home)*. In verb-final languages such as Japanese and Korean, however, long phrases tend to shift in front of shorter phrases (Yamashita and Chang, 2001; Dennison, 2008), showing a long-before-short preference. Thus, if the object (O) is modified by a long and semantically rich RC, the whole NP is likely to be scrambled to the sentence-initial position, resulting in an OSV structure. Using an L2 production task, Dennison (2008, p. 7) found that when the object was modified by a long RC, Korean natives and Korean-dominant bilinguals showed reduced preference for the canonical SOV order by shifting to the OSV order, producing 29.4 and 41.9% OSV sentences, respectively. In contrast, balanced and English-dominant bilinguals produced significantly less OSV sentences (only 6.8 and 3.3%, respectively), suggesting that they had a hard time switching from the L1 to the target L2 strategy. This study suggests that even though uttering a longer constituent first is arguably more cost-effective in forming long-distance (verb-argument) dependency in SOV languages (Hawkins, 2004, p. 108), bilinguals can still be heavily influenced by their entrenched L1 processing strategies.

However, given that only bilinguals of Korean and English were examined in Dennison (2008), it is not clear to what extent her conclusion is generalizable to late adult L2 learners whose L1s are (dis)similar to the target (L2) language in more than one aspect. Building on the finding of Dennison (2008), we aim to contribute to this line of L2 research by studying online sentence production patterns of a syntactically flexible structure in Chinese.

In Chinese, a demonstrative-classifier (DCL) phrase can precede or follow an RC, yielding either a DCL-first configuration (i.e., DCL-RC-HN) or a DCL-second configuration (i.e., RC-DCL-HN).^1^ See below an example for each configuration, one in subject-extracted RC [SR, ex. (1)] and the other in object-extracted RC [OR, ex. (2)], where the head noun “bicycle” is extracted from the subject or the object position of the RC, respectively, leaving a co-indexed trace (marked by t_*i*_) behind (Aoun and Li, 2003). Note that *liang* is the classifier denoting vehicles.

(1) DCL-first configuration in subject relative clause (SR) in Chinese

**na-liang** [_SR_ t_i_ zhuangdao luren de] zixingche_i_ xuyao xiuli

that-CL_vehicle_ _ hit-down pedestrian DE bicycle need repair

“The bicycle that hit the pedestrian needs repairing.”

(2) DCL-second configuration in object relative clause (OR) in Chinese

[_OR_ luren tuidao t_i_ de] **na-liang** zixingche xuyao xiuli

pedestrian knock-over _ DE that-CL_vehicle_ bicycle need repair

“That bicycle which the pedestrian pushed down needs repairing.”

While both configurations are allowed in Chinese grammar, existing work using corpora (Tang, 2007; Ming, 2010; Wu et al., 2012; Sheng and Wu, 2013) and online sentence production (Wu and Sheng, 2014) has suggested that in actual usage, the positioning of the DCL is contingent upon the type of RC. Specifically, native speakers of Chinese tend to place the DCL before an SR [i.e., short-before-long preference, ex. (1)], but after an OR [i.e., long-before-short preference, ex. (2)]. This asymmetric pattern of DCL positioning by RC types differs from both the short-before-long preference in English on the one hand and the long-before-short preference in Japanese and Korean on the other. Thus, it will be of theoretical interest to investigate whether English-, Japanese-, and Korean-speaking L2-Chinese learners can acquire the native-like asymmetric pattern.

### Processing-Driven Account for the Asymmetric Pattern in Chinese

Before investigating to what extent L1 processing strategies impact L2 production patterns, we need to first of all understand what underlies this intriguing asymmetric pattern in Chinese. Here we focus on a processing-driven account (Wu, 2009; Wu and Sheng, 2014; Wu et al., 2018), attributing Chinese native speakers’ production preference to computational efforts involved in using the classifier cue in DCL to build the complex S/OR structure during incremental processing. Consider uttering the SR. As required by Chinese grammar, a classifier is obligatorily congruent with the head noun of the RC. The early presence of classifier in (1) flags an upcoming, semantically matching noun, thus helping the speaker to retrieve the head noun from her mental lexicon much earlier compared to its late occurrence (i.e., the DCL follows the SR), and to build the complex RC structure itself. Furthermore, deferring constituents that are long and complex would buy speakers more time for structural formulation. Since DCLs are morpho-syntactically less weighty than RCs in terms of phrasal length and structural complexity, uttering the DCL first conforms to the accessibility principle or the short-before-long preference widely attested in SVO languages like English (Wasow, 1997; Clark and Wasow, 1998; Stallings et al., 1998; Arnold et al., 2000). In short, uttering DCLs first can relieve Chinese speakers of the pressure to plan the complex SR.

In the case of ORs, however, the picture gets more complicated when one utters DCLs first. Suppose the DCL *na-liang* in (2) is placed at the left edge of the OR, immediately adjacent to the OR-subject (*luren* “pedestrian”). Because activation of the classifier typically prompts Chinese speakers to retrieve a semantically congruent noun (Hsu et al., 2006, 2014; Zhou et al., 2010; Qian and Garnsey, 2016; Wu et al., 2018), whereas the vehicle-denoting classifier *liang* is incompatible with human referents, such a local classifier-noun mismatch (*na-liang luren*…) would impede lexical access and incur disruption during incremental sentence production. It is certainly an option for the speaker to maintain the congruent head noun in working memory and to suppress competition from the OR-subject while building the OR. But doing so would put a high demand on cognitive resources (Hsu et al., 2014). Thus, to avoid disruption of lexical access and to lessen the cognitive burden, Chinese native speakers tend to postpose the DCL in the case of OR.

### Cross-Linguistic Variations in the Positioning of Determiner Phrases in Relative Clauses

The asymmetric positioning of DCLs by RC type reflects Chinese native speakers’ processing strategies associated with classifiers in planning complex RC structures. However, given that languages vary in lexical categories and processing strategies, it is important to investigate whether L2 learners of Chinese can utilize the classifier cue in a native-like manner. We chose to study three groups of adult learners whose native languages are English, Japanese, and Korean because these languages vary in at least two aspects regarding the structure of a determiner phrase (DP) co-occurring with an RC. First, in terms of head direction, English has postnominal RCs, as is typical of languages with VO word order, whereas Korean and Japanese have prenominal RCs, as is typical of OV languages (Greenberg, 1963; Dryer, 2011). Second, in terms of the functional category of determiner (D), English only has (i) the article system that specifies (in)definiteness of the noun and (ii) the number morphology that marks the countability of the noun, without the category of classifier^2^ (CL) that is unique to classifier languages, whereas Japanese and Korean as East Asian languages, just like Chinese, are numeral-classifier languages (Gil, 2013). Given the similarities of Japanese and Korean to Chinese regarding these two parameters, a question immediately arises: Would it be easier for Japanese- and Korean-speaking learners of Chinese to acquire native-like production strategies than English-speaking learners? Here native-like performance means that attentional resources will be allocated differently depending on the function of classifier as a cue, both in (i) signaling an upcoming head noun, thereby yielding the target DCL-first order in SRs, and in (ii) avoiding potential lexical disruption in pre-OR positions, thus yielding the target DCL-second order in ORs.

However, despite the above similarities regarding the target structures *per se*, Korean and Japanese differ from Chinese in specific details regarding the realization of D when it co-occurs with RCs. One is the availability of D + CL combination. While Korean and Japanese are obligatory numeral-classifier languages, they only have N(umber)CLs but no D(emonstrative)CLs (Cui, 2014). To introduce deictic information to an NP modified by an RC, bare demonstratives—without the presence of classifiers—are used, specifically, *ku* (“that”) in Korean and *a-no* (“that”) in Japanese. In other words, bare demonstratives in Korean- and Japanese-RCs are the equivalent of DCLs in Chinese RCs.

The second difference concerns the positioning bias of demonstratives in RCs. While in theory positioning of demonstratives is flexible in Japanese and Korean [see examples of SR in (3–4)], the D-second configuration is likely to be preferred over the D-first configuration, due to (i) the long-before-short preference in OV languages (Hawkins, 1994; Yamashita and Chang, 2001) and (ii) avoidance of local ambiguity or difficulty in lexical retrieval (e.g., *ano hito*. in Japanese, or *ku hayngin*…in Korean, both meaning “that pedestrian…”). Indeed as revealed by a recent Chinese-Japanese translation corpus study, a general post-RC bias of demonstratives was found in Japanese (Sheng, 2010). Similarly, Kim (2014, p. 95) argued that in Korean, a non-restrictive RC (fully fledged or reduced) always precedes the demonstrative.

(3) Japanese SR with flexible positioning of demonstratives

(a-no) [_*SR*_ t_*i*_ hito-ni butuk-katta] (a-no) jitensya_*i*_-wa syuurisuru-beki-da

that _ pedestrian-ACC hit-PAST that bicycle-TOP repair-should-DECL

“That bicycle which ran into the pedestrian needs repairing.”

(4) Korean SR with flexible positioning of demonstratives

(ku) [_*SR*_ t_*i*_ hayngin-ul chi-n] (ku) cacenke_*i*_-nun swuli-ka philyoha-ta

(that) _ pedestrian-ACC hit-COMP that bicycle-TOP repair-NOM need-DECL

“That bicycle which ran into the pedestrian needs repairing.”

We summarize the similarities and differences between target L2-Chinese and the three languages in Table 1.

**TABLE 1:** Summary of similarities and differences between target L2-Chinese and L2 learners’ native languages.

**Parameter**	**L2 learners’ native languages**			**Target L1 Chinese**
	**English**	**Japanese**	**Korean**	
Canonical word order	SVO	(S)OV	(S)OV	(S)VO
Direction of RC	Postnominal	Prenominal	Prenominal	Prenominal
Classifier language	X	**√**	**√**	**√**
DCL in RC	X	X	X	**√**
Position of DPs in RC	Det-HN-RC	Postnominal bias	Postnominal bias	asymmetric

### Previous L2 Studies on Demonstrative-Classifier Positioning in Chinese Relative Clauses

While an increasing number of studies have shown that when uttering RCs, native speakers of Chinese favor the DCL-first configuration in SRs and the DCL-second configuration in ORs, relatively little is known regarding how adult L2 learners position DCLs when producing Chinese RCs. To our knowledge, existing work on DP positioning in L2-Chinese RCs has almost exclusively focused on English-speaking learners (Xu, 2009; Li J., 2013; Wu and Sheng, 2014), with few on Japanese-speaking (Lyu and Wu, 2017) or Korean-speaking (Wu and Lyu, 2016) learners. However, none of them directly compared differences across different groups using inferential statistical methods. Furthermore, the results are rather mixed, and even puzzling.

Consider the production experiments first. Using an offline task of filling in the blank with given words, Xu (2013) found that American intermediate learners of Chinese showed no particular bias for DCL positions when completing 347 sentences with SRs (55.62% DCL-first vs. 44.38% DCL-second), but a bias for the DCL-second configuration (62.26%, in contrast to 37.74% DCL-first configuration) when completing 204 sentences with ORs.^3^ However, using phrase-based production task, Wu and Sheng (2014) found that advanced English-speaking learners showed an overall bias for the DCL-first configuration when uttering both SRs (123/148, 83.11%) and ORs (122/193, 63.21%). Clearly, these two experimental studies yield inconsistent patterns for L2 learners whose L1 is English. Note, however, all the DCLs in Xu (2013) contained the generic classifier *ge*, which, bleached of semantic uniqueness, can also be used to modify the immediately following OR-subject, thus forming a local DP whose literal interpretation (e.g., “the boy who **that girl** loves”) is not the intended meaning (“**that boy** who the girl loves”), as in *[_*OR*_ na-ge_*i*_ nvehai aishang de nansheng_*i*_] “that-CL girl love REL boy.”* This ambiguity would render the classifier cue ineffective in predicting the RC structure. In the current study, we followed Wu and Sheng (2014) by using non-generic classifiers.

Regarding L2 learners whose L1s are OV languages, Japanese speakers were found to pattern like native speakers of Chinese by producing the asymmetric pattern, specifically, 154 DCL-1st vs. 104 DCL-2nd in SRs and 119 DCL-1st vs. 152 DCL-2nd in ORs (Lyu and Wu, 2017). However, Korean speakers were found to have no particular bias (142 DCL-1st vs. 176 DCL-2nd) in SRs, but a bias for the DCL-second configuration (175, in contrast to122 DCL-1st) in ORs (Wu and Lyu, 2016). Note that these two studies used exactly the same procedure (i.e., phrase-based production paradigm), thus rendering the distinct patterns rather puzzling, assuming that Japanese and Korean are typologically similar and share a number of properties with their L2-Chinese in terms of the target structure.

In contrast to the inconsistent findings in prior sentence production work, the patterns yielded by existing L2 corpus studies appear to be quite enlightening. Using the inter-language composition corpus of the Chinese Proficiency Test (HSK), these studies examined the distributional patterns of classifiers (both DCLs and NCLs) in RCs produced by intermediate and advanced L2-Chinese learners (English: Li J., 2013; Korean: Wu and Lyu, 2016; Japanese: Lyu and Wu, 2017). We summarize their findings in Table 2.

**TABLE 2:** Distribution of NCL/DCLs in RCs found in the inter-language composition corpus reported in existing works on English-, Japanese-, and Korean-speaking L2-Chinese learners.

**L2 learners**	**L1-English** (Li J., 2013)		**L1-Japanese** (Lyu and Wu, 2017)		**L1-Korean** (Wu and Lyu, 2016)	
	**SR**	**OR**	**SR**	**OR**	**SR**	**OR**
	**# (%)**	**# (%)**	**# (%)**	**# (%)**	**# (%)**	**# (%)**
DCL/NCL-1st	42 (91.30%)	7 (58.33%)	27 (84.38%)	15 (100%)	25 (80.65%)	6 (35.29%)
DCL/NCL-2nd	4 (8.70%)	5 (41.67%)	5 (15.63%)	0 (0%)	6 (19.35%)	11 (64.71%)
Total	46 (100%)	12 (100%)	32 (100%)	15 (100%)	31 (100%)	17 (100%)

First, regardless of their L1s, L2 learners generally preferred the DCL/NCL-first configuration in SRs (English: 42/46, 91.30%; Japanese: 27/32, 84.38%; Korean: 25/31, 80.65%). Second, in the case of ORs, English L2-Chinese learners showed no particular bias for DCL/NCL positioning, whereas Korean and Japanese L2-Chinese learners appeared to have a bias for DCL/NCL-second configuration in ORs. However, it is worth noting that in contrast to an overall high production rate of SRs, L2 learners produced few tokens of ORs (English: 12; Japanese: 15; Korean: 17). Recall that the DCL-OR order is computationally demanding even for native speakers of Chinese, thus these L2 learners of Chinese were likely to strategically avoid producing DCLs that co-occurred with ORs in off-line writing tasks.

To summarize, while the findings from L2-learners’ composition-based corpora are quite revealing in how L1s were potentially at work in off-line sentence production, the small size of target OR structures in the corpora could render statistical analyses difficult. Thus, lab-controlled experiments might be a viable way to further examine L2 productions. However, existing experimental work only examined a particular L2 group, and the statistical analyses were conducted using chi-square tests. All this renders it difficult to make cross-group comparisons. In view of inconsistent and puzzling findings of phrase-based production experiments, we note that advanced L2 participants might vary in their proficiencies, as reflected by the low production rates of “completely correct” RCs in English speakers (Wu and Sheng, 2014, p. 406, Table 1), with 56.27% accuracy in SRs and 73.11% accuracy in ORs. Thus, we aimed to use the same experimental procedure as in prior work, while controlling the potential proficiency confound, and to further investigate whether adult L2 learners whose L1 processing strategies vary typologically could produce in real time the target-like asymmetric pattern in Chinese RCs.

## Research Questions and Predictions

We set out to probe the online production patterns of DCL positioning in Chinese RCs by advanced L2 learners whose L1s are English, Japanese, and Korean. We ask the following research questions:

(i) Can advanced L2-Chinese learners with different L1 backgrounds acquire native-like processing strategies, specifically, favoring the DCL-first configuration in SRs and the DCL-second configuration in ORs? If not, how deviated are they from native speakers in terms of their production patterns?

(ii) Are there any differences in production patterns between L2 groups? Specifically, how do Korean and Japanese natives differ from English natives? In addition, do Korean and Japanese natives differ despite that they both reportedly prefer the long-before-short strategy?

We remain agnostic about the prediction of the first question due to (i) its empirical nature and (ii) the mixed results from existing work. Regarding the second question, as determiners always precede other nominal modifiers in English, assuming L1 transfer, we predict that English L2-learners are more likely to prepose the DCL compared to Japanese and Korean groups whose L1s exhibit a long-before-short preference. Alternatively, with sufficient language experience, English learners could override L1 influence and do not show any difference compared to Japanese and Korean learners. Regarding the second half of the question, building on existing corpus findings (Wu and Lyu, 2016; Lyu and Wu, 2017), we predict that with proficiency being well controlled, Korean and Japanese natives are expected to pattern alike, and possibly approximate native-like asymmetric patterns.

## The L2 Production Experiment

To answer empirical questions and to test our predictions, we adopted the same experimental procedure as in existing work (Wu and Sheng, 2014; Wu and Lyu, 2016; Lyu and Wu, 2017), namely, a phrase-assembly task that is commonly used in the sentence production literature (Ferreira, 1996; Yamashita and Chang, 2001; Dennison, 2008; Hwang and Kaiser, 2015).

### Methods

#### Participants

We recruited 47 English, 37 Korean, and 38 Japanese native speakers who were at intermediate-high or high levels of Chinese proficiency from universities in Shanghai, Beijing and Nanjing. All had either passed the standardized Chinese Proficiency Test (HSK^4^). Level 5/6 by the time they participated in the experiment or had reached the high-proficiency level by taking advanced Chinese classes. Prior to the production experiment, all L2 participants were required to take a screening test. The test consisted of 40 words from the HSK (Level 6) vocabulary list, all to appear in the production experiment. Participants were shown each word on a computer screen and were asked to read it aloud. Only those whose initial accuracy rate exceeded 70% were allowed to proceed, resulting in 37 English (mean age = 23.3 years, *SD* = 2.68, mean years of studying in China = 4.1), 35 Korean (mean age = 23.7 years, *SD* = 4.18, mean years of studying in China = 7.3), and 37 Japanese (mean age = 21.2 years, *SD* = 2.43, mean years of studying in China = 4.3) L2 learners. They then received intensive training on those words, until they recognized all the words in a follow-up test.

Twenty-two native speakers of Chinese (mean age = 21.8 years, *SD* = 1.4) from a university in Shanghai also participated in the experiment as the control group.

#### Materials and Design

##### Written Stimuli

Experimental stimuli consisted of 24 sets of Chinese sentences, each in two conditions: SR or OR, as in (5a-b). Each sentence was chunked into four parts: RC, DCL, HN, and MC. To necessitate the use of individual classifiers, all HNs were inanimate, and RC-internal NP animate.^5^ DCLs matched the inanimate HN only, and mismatched the RC-internal NP, thus ruling out the confound in Xu (2013) where the generic classifier *ge* can modify both the RC-internal NP and the HN.

(5) A sample set of stimulus in four chunks (written in Chinese characters in actual experiment):
  a. | _*SR*_ hit-down pedestrians DE | DCL_*i*_ | _*HN*_ bicycle_*i*_ | _*MC*_ need repairing |
  b. | _*OR*_ pedestrians push-down DE | DCL_*i*_ | _*HN*_ bicycle_*i*_ | _*MC*_ need repairing |

We also created 36 fillers of various constructions,^6^ all chunked into four parts. The target items were combined with the fillers to form 2 counterbalanced lists. Thus, each list contained 24 targets and 36 fillers, which were then pseudo-randomized such that no more than two target sentences appeared in succession.

##### Visual Display

**FIGURE 1:**
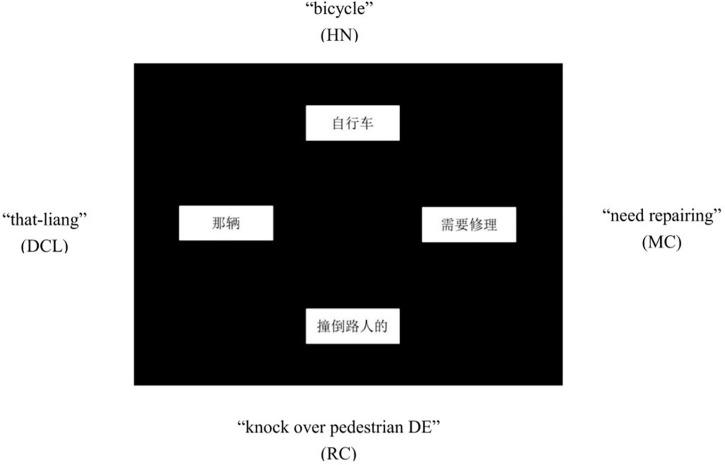
An example of the visual display.

For each sentence, the four chunks (i.e., DCL, HN, RC, MC) were pseudo-randomly assigned to four rectangular boxes that appeared in fixed positions on the computer screen: top, left, right, and bottom (Figure 1).

As our primary concern was where exactly in participants’ utterances the DCL was located relative to the RC and the HN, we selected the DCL as a reference point, and assigned the RC and the HN next to its sides to form a triangle, leaving the fourth box to the MC. To minimize potential effects of physical distance on participants when they conceptualized or planned utterances, we kept the visual distance between DCL and RC the same as the distance between DCL and HN. This design yielded 8 possible visual displays. DCL on the top, however, would have resulted in HN and RC right below it (left or right), yet the linear presentation of these words might lead a “visual” participant to simply read them out, producing the target DCL-RC order (e.g., “DCL knock over pedestrians DE”) or a DP sequence of DCL-HN (e.g., “that girl”). To eliminate this positioning confound and to make the task more challenging, these two versions were excluded, leaving 6 visual layouts in total for further counterbalancing.

#### Procedure

**FIGURE 2:**
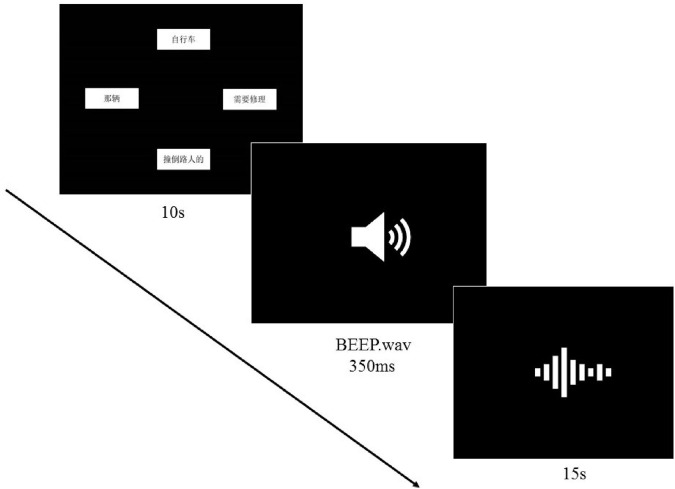
Experimental procedure for L2-Chinese learners.

The visual stimuli were presented by Paradigm (Perceptual Research System Inc.). Instructions were written in participants’ native languages to ensure that participants understood the task. Participants were seated in front of a laptop in a quiet room wearing a Sennheiser headphone. They pressed the space bar to initiate the trial. For each trial, participants viewed the visual stimuli in four chunks. Their task was to combine the four fragments presented on the screen into a sentence that sounded natural to them, and to utter it when they were ready. In 10 s they heard a 350 ms-long beep, upon which they had to speak out if they had not yet done so. They had 15s to finish the sentence, or the visual stimuli disappeared. Thus, each trial lasted 25 s. Figure 2 illustrates the experimental procedure.

To make the task more engaging for native speakers of Chinese, the visual stimuli vanished from the screen in 4,500 ms, instead of always remaining in sight for L2 participants. Meanwhile, the beep sound was presented.

All participants had four practice trials to familiarize themselves with the procedure. They took a rest when finishing half of the trials. The whole experiment took approximately 30 min.

#### Data Coding

Participants’ utterances were categorized into three types based on grammaticality and expectedness:

**Type 1: Target utterance**. Participants used all the four components on the screen, producing grammatical sentences as expected, as in (1).

**Type 2: Grammatical but unexpected**. The utterances were grammatical but deviated from expected forms, mainly due to position exchange or omission of components.

**Type 3: Ungrammatical**. The utterances were incomplete sentence fragments, syntactically ill-formed, or semantically anomalous.

### Results

Data from 37 participants were eliminated from data analyses for the following reasons: (1) Ten participants (English: 6; Japanese: 4) had an extremely low rate of target utterances (cutoff being 50% for English, and 62.5% for Japanese/Korean); (2) Eleven participants (English: 5; Japanese: 4; Korean: 2) exclusively produced either a DCL-first or a DCL-second configuration (see Ferreira and Yoshita, 2003; Slevc, 2011 for similar practice); (3) Two participants (English: 1; Japanese: 1) self-reported in post-experimental interviews and were verified by their actual production that they adopted specific strategies in assembling chunks (e.g., always uttered the predicate first). In addition, given that the L2-Chinese proficiency was high for the Japanese and Korean natives overall compared to English natives, to ensure each group had comparable target structures (i.e., Type 1 utterances) for inferential statistics, we further eliminated fourteen participants (Japanese: 5; Korean: 9) who produced no more than two instances of DCL-first or DCL-second configuration. The remaining data of 94 participants were entered into statistical analyses, including 25 English, 23 Japanese, 24 Korean L2-Chinese learners and 22 native controls.

Table 3 shows the distribution of 3 types of utterances produced by the Chinese controls and the three groups of L2-Chinese learners.

**TABLE 3:** Utterance types produced by all groups of participants.

**Utterance type**	**Chinese controls (*N* = 22)**		**English learners (*N* = 25)**		**Japanese learners (*N* = 23)**		**Korean learners (*N* = 24)**	
	**SRC # (%)**	**ORC # (%)**	**SRC # (%)**	**ORC # (%)**	**SRC # (%)**	**ORC # (%)**	**SRC # (%)**	**ORC # (%)**
Target utterance	241(91.3)	228(86.4)	221 (73.7)	251 (83.7)	240 (87.0)	252 (91.3)	257 (89.2)	268 (93.1)
Grammatical but unexpected	14 (5.3)	24 (9.1)	12 (4.0)	4 (1.3)	9 (3.3)	7 (2.5)	5 (1.7)	2 (0.7)
Ungrammatical	9 (3.4)	12 (4.5)	59 (19.7)	30 (10.0)	23 (8.3)	17 (6.2)	24 (8.3)	14 (4.9)
No response	0	0	8 (2.7)	15 (5.0)	4 (1.4)	0	2 (0.7)	4 (1.4)
SUM	264 (100)	264 (100)	300 (100)	300 (100)	276 (100)	276 (100)	288 (100)	288 (100)

Two observations are noteworthy. First, the accuracy rates of the target RCs were high overall (native controls: 88.8%; Korean: 91.1%; Japanese: 89.1%), except for the English group (78.7%).^7^ While it is possible that our English learners were less proficient than Korean and Japanese learners, this possibility was kept minimal as we used very strict criteria to screen L2 participants, and cares were taken to ensure that our L2 groups’ production rates of target utterances were comparable. We instead attribute the relative low accuracy rate of the English group possibly to head-direction in L1, which plays a role in modulating the ease of production. As English RCs are head-initial, it is conceivably more difficult for English-speaking learners of Chinese to produce head-final RCs compared with Korean and Japanese learners.

Second, L2 groups appeared to differ from native controls in RC accessibility. Consistent with the Noun Phrase Accessibility Hierarchy (Keenan and Comrie, 1977), Chinese speakers were numerically more accurate in uttering SRs than ORs (91.3% vs. 86.4%). In contrast, all three L2 groups appear to be more accurate in OR than in SR conditions (English: 83.7% vs. 73.7%; Korean: 93.1% vs. 89.2%; Japanese: 91.3% vs. 87.0%), but the difference did not reach significance. In fact, this numerical trend for OR advantage echoes existing L2-production studies (Wu and Sheng, 2014; Wu and Lyu, 2016; Lyu and Wu, 2017), possibly because the *NV*…sequence in the OR resembles the canonical word order (SVO) in Chinese.

Given that our study is mainly concerned with whether L2 learners could acquire native-like positioning patterns of the DCL, in the sections below we focus on *target* utterances only.

#### Distribution of Demonstrative-Classifiers in Target Relative Clauses

**FIGURE 3:**
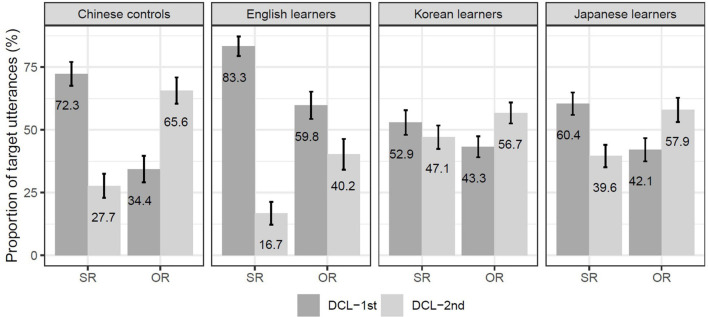
The distribution of DCL in SR and OR across Chinese controls and three L2 groups. Error bars are standard errors over by-participant means.

Figure 3 shows the distribution of DCLs as a function of RC types across the Chinese control group and three L2 groups. As revealed by the leftmost bars, the native control group produced an asymmetric pattern of DCL by the RC type. Regarding the three L2-learner groups, while the English group appears to show a general bias for the DCL-first configuration regardless of the RC type, the Korean and Japanese groups seem to pattern like the native control group, especially the Japanese group (see the rightmost bars).

#### Statistical Analyses

All *target* utterances were analyzed using logistic mixed-effects regression models in R using the *lme4* package (Bates et al., 2015), with DCL position (DCL-first = 1, DCL-second = 0) as the dependent variable. For all analyses, the initial model included a maximal random-effects structure that included all fixed effects and interactions among them. If the maximal model failed to converge, the random-effects structure was simplified by removing the random slopes of the fixed effects one at a time (Barr et al., 2013).

To check whether the four groups varied in their RC production patterns reflected by an interaction of *RC Type* × *Native Language*, we first built a full model with the fixed effects of *RC Type* and *Native Language* and their interactions, and then compared it with a model that excluded the interaction, using likelihood ratio tests (Barr et al., 2013). The result showed that the full model has a significantly better fit for the data than the model without the interaction [likelihood ratio test: *x*^2^(3) = 29.30, *p* < 0.0001], confirming that L2 groups’ production patterns were significantly deviated from those of the native controls.

To fully capture each participant group’s production patterns, we then fitted models for each group separately. RC Type (treatment-coded; baseline: OR) was included as the fixed effect. The model results of each group are summarized in Table 4. In the output, a positive log-odds coefficient means a bias for the DCL-first configuration, whereas a negative coefficient indicates a bias for the DCL-second configuration. For example, a positive log-odds coefficient for the Chinese control group in the SR shows that they were more likely to produce a DCL-first configuration in response to SRs.

**TABLE 4:** Logistic mixed-effects models by participant group and RC type including all perfect utterances.

	**Condition**	**Estimate**	**SE**	***t*-value**	***p*-value**
Chinese controls	SR	1.07	0.30	3.56	0.0004***
	OR	–0.87	0.32	–2.68	0.007**
English learners	SR	2.08	0.35	5.89	< 0.0001***
	OR	0.46	0.31	1.48	0.14
Korean learners	SR	0.18	0.25	0.71	0.48
	OR	–0.34	0.25	–1.34	0.18
Japanese learners	SR	0.46	0.21	2.22	0.03*
	OR	–0.36	0.20	–1.76	0.08

*Formula in R: dclPos* ∼ *rcType* + *(1* + *rcType | subject)* + (1 | item). ***p < 0.001; **p < 0.01; *p < 0.05.

Consistent with existing L1 literature, the native control group showed a significant asymmetric pattern of DCL by the RC type (SR: β = 1.07, SE = 0.30, *z* = 3.56, *p* < 0.001; OR: β = −0.87, *SE* = 0.32, *z* = −2.68, *p* < 0.01). Regarding the L2-learners, while the English group appeared to show a general bias for the DCL-first configuration, statistical significance was found in SRs only (β = 2.08, *SE* = 0.35, *z* = 5.89, *p* < 0.0001), but not in ORs (β = 0.46, *SE* = 0.31, *z* = 1.48, *p* > 0.05). The other two L2-groups seemingly behaved like native controls, but the Korean group only showed numerical trends for the asymmetric pattern (SRs: β = 0.18, *SE* = 0.25, *z* = 0.71, *p* > 0.05; ORs: β = −0.34, SE = 0.25, *z* = −1.34, *p* > 0.05), and the Japanese group showed a significant DCL-first bias in SRs only (SRs: β = 0.46, *SE* = 0.21, *z* = 2.22, *p* < 0.05; ORs: β = −0.36, *SE* = 0.20, *z* = −1.76, *p* > 0.05).

To specifically address our research questions, we further conducted statistical analyses for cross-group comparisons. To find out how L2 groups deviated from the Chinese native controls in their production patterns, logistic mixed-effects models were performed with DCL position as the dependent variable, and RC Type (treatment-coded; baseline: OR), Native Language (treatment-coded, baseline: Chinese) and their interactions included as fixed effects. To further probe how Korean and Japanese learners differed from English learners in their production pattern, we performed the same model, but this time set English as the reference level for Native Language. Similarly, to assess whether Korean learners deviated from Japanese learners in their utterance pattern, we reran the model with the Japanese group as the reference level. The model results are present in Table 5. We discuss each set of results below.

**TABLE 5:** Logistic mixed-effects models by group comparison and RC type including all perfect utterances.

**Group comparison models**	**Contrast**	**Estimate**	**SE**	***t*-value**	***p*-value**
L2 learners vs. Chinese controls	SR (English vs. Chinese)	0.95	0.39	2.47	0.01*
	SR (Korean vs. Chinese)	–0.83	0.36	–2.34	0.02*
	SR (Japanese vs. Chinese)	–0.54	0.36	–1.50	0.13
	OR (English vs. Chinese)	1.26	0.36	3.46	0.0005***
	OR (Korean vs. Chinese)	0.46	0.36	1.28	0.20
	OR (Japanese vs. Chinese)	0.43	0.36	1.19	0.23
Korean/Japanese vs. English learners	SR (Korean vs. English)	–1.79	0.38	–4.73	< 0.0001***
	SR (Japanese vs. English)	–1.49	0.38	–3.92	< 0.0001***
	OR (Korean vs. English)	–0.79	0.35	–2.29	0.02*
	OR (Japanese vs. English)	–0.82	0.35	–2.34	0.02*
Korean vs. Japanese learners	SR (Korean vs. Japanese)	–0.29	0.31	–0.94	0.35
	OR (Korean vs. Japanese)	0.03	0.31	0.10	0.92

*Formula in R: dclPos* ∼ *rcType* + *Native Language* + *rcType: Native Language* + *(1 | subject)* + (1 | item). ***p < 0.001; *p < 0.05.

#### L2 Groups vs. Chinese Controls

In Table 5, the results in the first row show how each L2 group deviated from the Chinese native controls. In the case of SRs, the English group showed a significantly stronger bias for the DCL-first configuration than the native controls (β = 0.95, *SE* = 0.39, *z* = 2.47, *p* < 0.05). In contrast, compared to the native controls, both the Korean and Japanese groups showed a stronger bias for the DCL-second configuration, but this bias reached significance only with the Korean group (β = −0.83, *SE* = 0.36, *z* = −2.34, *p* < 0.05), not with the Japanese group (β = −0.54, *SE* = 0.36, *z* = −1.50, *p* > 0.05).

In the case of ORs, compared to the native controls, the English group showed a significant bias for the DCL-first configuration (β = 1.26, *SE* = 0.36, *z* = 3.46, *p* < 0.0001), but neither the Korean nor the Japanese groups showed a stronger DCL-first bias (Korean: β = 0.46, *SE* = 0.36, *z* = 1.28, *p* > 0.1; Japanese: β = 0.43, *SE* = 0.36, *z* = 1.19, *p* > 0.1).

#### English Group vs. Korean/Japanese Groups

In Table 5, the second row reports the results of comparisons between English learners (whose L1 favors the short-before-long strategy) and Korean/Japanese learners (whose L1s prefer the long-before-short strategy), with English group as the reference level. In the case of SRs, both the Korean and Japanese groups showed a significant bias for the DCL-second configuration compared to English group (Korean: β = −1.79, *SE* = 0.38, *z* = −4.73, *p* < 0.0001; Japanese: β = −1.49, *SE* = 0.38, *z* = −3.92, *p* < 0.0001). This stronger bias for the DCL-second configuration was also found in the case of ORs for both the Korean and Japanese groups (Korean: β = −0.79, *SE* = 0.35, *z* = −2.29, *p* < 0.05; Japanese: β = −0.82, *SE* = 0.35, *z* = −2.34, *p* < 0.05).

#### Korean Group vs. Japanese Group

The last row of Table 5 reports the results of comparisons between the L2-learner groups whose L1s favor the long-before-short strategy. Compared to the Japanese group, the Korean group did not differ significantly in both SRs (β = −0.29, *SE* = 0.31, *z* = −0.94, *p* > 0.05) and ORs (β = 0.03, *SE* = 0.31, *z* = 0.10, *p* > 0.05).

## Discussion

Using a phrase-assembly task, we investigated the positioning of DCLs in Chinese RCs produced by advanced learners of Chinese whose native languages are English, Japanese, and Korean. Specifically, we examined how L1 production strategies affect L2 acquisition of the target structure alternations (i.e., DCL-SR and OR-DCL). We obtained four major findings. First, in terms of DCL positioning in SRs, compared to the Chinese natives, the English group showed an even stronger bias for the DCL-first configuration, whereas the Korean group showed a stronger bias for the DCL-second configuration, with the Japanese group approximating native-like performance. Second, in terms of DCL-positioning in ORs, compared to the Chinese natives, the English group still showed a stronger bias for the DCL-first configuration, but both the Korean and Japanese groups approximated native-like performance. Third, compared to the English group, the Korean and Japanese groups favored the DCL-second configuration in both SRs and ORs. Fourth, no differences were found between the Korean and Japanese groups. As discussed below, these findings suggest that L1 processing strategies play a deterministic role in modulating L2 acquisition of native-like production strategies, at least in the specific construction of DCL positioning in Chinese RCs.

### English Group: Lack of Classifier Weakens Sensitivity to Lexical Disruption in DCL-OR

Compared to the native controls’ asymmetric patterns of DCL positioning by RC type, the English group showed a stronger bias for the DCL-first configuration in both SRs and ORs, suggesting that the short-before-long processing strategy in their L1 plays an overarching role. Their native-like bias for the target DCL-SR order suggests that despite the fact that English RCs are postnominal, resetting the parameter of head-direction to prenominal RCs does not impose much difficulty to late adult learners. Rather, it is the lack of the category of classifier in L1-English that affects their ability to fully produce the target OR-DCL order.

The category of classifier in Chinese is perhaps more complex than merely a functional category as the generative grammar labels it (e.g., Cheng and Sybesma, 1999, 2012; Huang et al., 2009; cf. Wu and Bodomo, 2009; Li X., 2013). It requires agreement to the noun it modifies, but the criteria for classifier-noun congruence are rather random, sometimes arbitrary (Greenberg, 1972; Gao and Malt, 2009). As reported in Hansen and Chen (2001), even highly proficient missionaries immersed in Chinese-speaking regions for years had problems with classifiers once they stopped receiving extensive input. Thus, to English-speaking late adult learners whose L1 does not encode this category at all, mastery of classifier-noun congruence necessarily involves vast exposure and considerable memorization, without which it would be difficult to sensitize them to the incongruence between a classifier and a following noun as in the case of DCL-OR. Furthermore, the OR-DCL order involves additional cognitive resources or computational steps compared with the DCL-OR order^8^ (Ming, 2010; Ming and Chen, 2010; Zhang, 2015). Recall that it is the local classifier-noun incongruence that impedes lexical retrieval and prompts Chinese speakers to postpose the DCL. The extra step of postposing DCLs certainly demands cognitive resources, whereas late adult L2-Chinese learners are known to have limited working memory capacity (for a meta-analysis, see Linck et al., 2013). Specifically, for our English group, using the classifier cue to construct a prenominal OR might have already been cognitively demanding, as it involves a long-distance dependency between the classifier and the clause-final head noun (Hsu et al., 2014; Qian and Garnsey, 2016), leaving little resource to compute or even “notice” the local incongruity between the classifier and its immediately following OR-subject. In short, the complexity underlying the target OR-DCL linearization, in conjunction with the lack of classifiers in L1, explains the English group’s overall bias for the DCL-first configuration.

Do English-speaking learners have a representation of classifiers at all? We believe the answer is affirmative. First of all, the DCL positioning differences in SRs vs. ORs suggests that English learners did make some effort, however minimal, to shift the DCL. Second, if due to lexical transfer English-speaking learners merely treated the DCL in Chinese as equivalent to the definiteness-denoting determiner in English, ignoring the classifier in the DCL in their representation of inter-language, then we would expect to see a large number of DCL-HN-RC (i.e., postnominal RC) utterances, given the convenience of forming an English version of a DP consisted of a determiner and a noun. But on the contrary, we only found 5.83% (35 tokens, including 19 *Ungrammatical* ones) of such structure, which is indeed allowed in spoken Chinese (Li and Thompson, 1984; Wang and Wu, 2020). Note, however, that this type of postnominal RC structure was also produced by Japanese (21 tokens, 3.80%) and Korean (7 tokens, 1.22%) speakers. Thus, we contend that for the English L2-Chinese learners, it is just the collocation of which classifier goes with which noun that goes beyond their learnability.

### Korean and Japanese Groups: Pattern Alike

Our Korean and Japanese groups pattern alike for both SRs and ORs, without any differences being found between them, in contrast to prior findings. Recall that one puzzling pattern of results in exiting work is that Koreans were found to show an overall preference for the DCL-second configuration in both SRs and ORs (Wu and Lyu, 2016), whereas Japanese L2-learners approximated native speakers’ asymmetric pattern of DCL positioning (Lyu and Wu, 2017). Note, however, these two works were presented as separate studies, unlike the current study that focused on between- or cross-group comparisons using modern statistical analyses. Furthermore, the current study had much stricter criteria in screening our L2 participants as advanced learners of Chinese, whose production rates of target utterances were of similar magnitude across the L2 groups. Thus, we believe our current findings are statistically valid and, crucially, logically sound, given that both languages are typologically similar, with specific parameters (i.e., head directionality, classifier) examined in the current study very much alike as well. Therefore, our finding that Korean and Japanese groups did not differ in positioning DCLs in different types of RCs fits our expectations.

Additional evidence for the Korean and Japanese groups to pattern alike comes from a unanimous DCL-second bias in SRs and ORs when their utterances were compared to those of the English group. Note that here the between-group comparisons were made on a relative scale, that is, when viewed separately, both Korean and Japanese group appeared to show a trend for the DCL-first bias in SRs, but the even stronger DCL-first bias demonstrated by the English group rendered them to be showing a DCL-second bias. Likewise, while both Korean (56.7%) and Japanese (57.9%) tended to put DCLs after ORs, this DCL-second bias was rendered even stronger by the much less production rate of the OR-DCL-HN structure of the English group (40.2%). Here we would like to argue that to both the Korean and Japanese groups, the strong “long before short” preference was very likely to bring additional benefits in helping them utter ORs, given that the target OR-DCL order potentially involves complex computation. Furthermore, known as numeral-classifier languages, Korean and Japanese has the category of Classifier. Thus, it might come as no surprise for those adult learners of Chinese to be sensitive to the local classifier-noun mismatch in the DCL-OR order, hence yielding more target OR-DCL structures than their English counterparts.

### Korean and Japanese Groups: Subtle Differences in Comparison With Native Controls

It is worth noting that the Korean group differed from the Japanese group in subtle aspects when their utterances were compared to those of the native controls. The Japanese group did not deviate from the native group, by producing the asymmetric pattern of DCL-positioning by RC types. But the Korean group showed a stronger bias for the DCL-second configuration in SRs than the native controls, while they approximated the native-like pattern in ORs.

Why then did the Korean group (47.1%) show a stronger DCL-second bias than the native controls (27.7%) when uttering SRs? Here we would like to argue that L1 and L2 processing strategies competed for determining the production patterns such that neither strategy gained an upper hand. Recall that Korean prefers the long-before-short strategy, rendering the D-RC configuration less preferred in L1-Korean than the RC-D configuration. For our Korean group, this language-specific constraint might neutralize the high accessibility of DCL (recall that DCL is shorter and syntactically less weighty than SR). This lends support to the Processibility Theory (Pienemann, 1998, p. 82) that “L1 procedures may be transferred when they are processable within the interlanguage system, i.e., as soon as the necessary prerequisites have been developed,” where L1 procedure includes linearization of “word order.” The fact that the RC-D-HN order is highly preferred than the D-RC-HN order and thus more processable in L1-Korean explains why the Korean-speaking learners were more likely to produce the target OR-DCL order in L2-Chinese, and the non-target SR-DCL order as well, when compared to the native controls. Our results from the Korean group are also consistent with the finding in Dennison (2008), showing that it is very difficult, if not impossible, to eradicate the long-before-short processing strategy in L1-Korean.

The fact that the Korean group diverged from the Chinese controls in SRs further suggests that while the Korean learners were aware of the processing advantage brought about by the DCL-SR configuration, they relied to a lesser extent on the classifier as a cue to plan speech, presumably influenced by the vestigial, however, minimal, long-before-short strategy. Now the question is whether this is the final stage of their L2 acquisition process or whether the Korean learners are still in the process of full attainment in a native-like manner. If it is the former, the current findings only demonstrate the deterministic role of L1 processing strategy in L2 production, but do not fully support the Unified Competition Model (UCM) (MacWhinney, 2005). If, however, the Korean learners are still on their way to internalizing the L2-Chinese processing strategies of DCL positioning, the current study might be a perfect case illustrating a transitional stage of L2 development, which is predicted by the UCM. To shed light on this issue, future studies could benefit by looking at Korean L2-Chinese learners who have prolonged exposure to Chinese in an L2 language environment.

Critically, unlike the Korean group, why were the Japanese group able to produce the target DCL-SR order, approximating the native-like performance? Given that the target DCL-SR order is not computationally demanding and that Japanese does encode Classifier, it might not be too surprising that Japanese-speaking adult learners with enough experience in their L1, if not enough usage in L2-Chinese, were able to use the classifier cue to help them utter the SR. This leads us to a somewhat different conclusion from Dennison (2008), which implied that L2 processing strategies are impossible to acquire. Notice that Dennison (2008) examined learners with only one linguistic background, namely Korean, yet there exist minute differences among native speakers and bilinguals with different proficiencies in Korean. Here we argue that L2 processing strategies can be acquired, as demonstrated by our data from the Japanese group. Our study also lends support to the UCM (MacWhinney, 2005), one essential claim of which is that when faced with options, L2 learners might necessarily transfer their L1 processing strategies *en masse* to the target L2, but it is not an insurmountable task to inhibit the L1 strategy and to switch to the L2 strategy. Through extensive naturalistic exposure to the target language, a gradual shift to the target-like production pattern will emerge. The fact that our Japanese group displayed an asymmetric DCL positioning pattern, instead of a DCL-second bias in SRs as their Korean counterparts did, argues in favor of this hypothesis at least to some extent.

## Conclusion

The present study is the first to investigate DCL positioning in spoken production across three learner groups with different typological encoding of determiner phrases. We found English L2 participants showed distinct patterns from Korean and Japanese L2 participants, but Korean and Japanese very much resembled each other. While these advanced L2 learners deviated in varying degrees from the native controls in attaining native-like production strategies, their overall performance was subject to L1 transfer. We conclude that when processing strategies compete between L1 and L2, native-like processing strategies can be acquired, yet the ultimate success is contingent upon L1-specific properties.

## Data Availability Statement

The raw data supporting the conclusions of this article will be made available by the authors, without undue reservation.

## Ethics Statement

The studies involving human participants were reviewed and approved by Institutional Review Board, School of Foreign Language, Shanghai Jiao Tong University. The patients/participants provided their written informed consent to participate in this study.

## Author Contributions

FW: conceptualization, funding acquisition, methodology, resources, supervision, validation, writing—original draft, and review and editing. JL: data curation, investigation, validation, writing—original draft, and review and editing. YS: data curation, formal analysis, investigation, methodology, software, validation, visualization, and writing and editing. All authors contributed to the article and approved the submitted version.

## Conflict of Interest

The authors declare that the research was conducted in the absence of any commercial or financial relationships that could be construed as a potential conflict of interest.

## Publisher’s Note

All claims expressed in this article are solely those of the authors and do not necessarily represent those of their affiliated organizations, or those of the publisher, the editors and the reviewers. Any product that may be evaluated in this article, or claim that may be made by its manufacturer, is not guaranteed or endorsed by the publisher.

## Footnotes

1 Existing research has been mixed regarding semantic interpretation of these two alternations. Some support a distinction of restrictive vs. non-restrictive reading (Chao, 1968; Yue-Hashimoto, 1971; Huang, 1982), whereas others do not (Del Gobbo, 2003, 2010; Lin, 2003; Zhang, 2006). The semantic distinctions between these two patterns, however, are not directly related to the major concern of the current paper, nor do they affect our conclusions.

2 While English has fairly productive measure words [e.g., “two kilos/pounds/boxes (of apples),” “a pair (of shoes),” “a pack (of people),” “a flock (of sheep)”], these “mensural classifiers” are different from the “sortal” or “individual” classifiers used in our production experiments. First, whereas mensural classifiers can be used for measuring units of conceptually count and mass nouns, individual classifiers are used only with conceptually count nouns (e.g., Croft, 1994, pp. 151–152; Cheng and Sybesma, 1999, p. 515). Second, constructions corresponding to the mensural classifier phrases are said to occur in all languages, whereas individual classifiers are unique to classifier languages only (e.g., Croft, 1994, p. 152; Lyons, 1977, p. 464; Löbel, 2000, p. 223).

3 Here we focus on target-like RC responses reported in Table 2 of Xu (2013, p. 178), leaving aside 13 RCs with errors. Out of 347 SRs, 193 (55.62%) occurred in the DCL-first condition, and 154 (44.38%) in the DCL-second condition. Out of 204 ORs, 127 (62.26%) were in the DCL-second condition, whereas 77 (37.74%) were in the DCL-first condition.

4 Effective in 2007, the new version of HSK assigns test-takers to 6 proficiency levels. Test-takers passing HSK levels 5-6 are considered as highly proficient in Chinese.

5 This means that the ORs had the typical animacy configuration (i.e., head noun = inanimate, embedded noun = animate), but the SRs had the reversed animacy configuration (i.e., head noun = inanimate, embedded noun = animate). Note, however, the seemingly unusual configuration in SRs is consistent with the finding of existing Chinese corpus studies, that is, while head nouns of ORs prefer to be inanimate, head nouns of SRs show no particular preference for animacy (Wu et al., 2012; Hsiao and MacDonald, 2013).

6 Filler items consisted of 10 reason-, manner-, time-, place, or instrument-adjunct RCs (i.e., without gaps), 13 sentences without RCs, and 13 simple sentences with different uses of the adnominal DE (adjective-marking: 4, genitive-marking: 5, noun-complement-marking: 4).

7 Note that if we count in the “Grammatical but Unexpected” utterances, the accuracy rate of the English group would be 81.3%.

8 For linguistic and functional accounts for the RC-DCL order being more complex than the DCL-RC order, see Ming and Chen (2010) and Zhang (2015), respectively. According to Zhang (2015), the DCL-first configuration is the underlying representation, from which the DCL-second configuration is derived. Zhang (2015)’s argument is supported by the corpus study by Ming and Chen (2010), which shows that the DCL-first configuration occurs more frequently than the DCL-second configuration. According to Ming (2010) and Chen et al. (2015), the DCL-first configuration is used with entities with high discourse saliency and serves the function of characterization, whereas DCL-second configuration mainly appears with entities of low saliency and serves the function of identification.
